# The Quest for Truth: Experimenter Identity Impacts Children’s Response to Surprising Information

**DOI:** 10.1162/opmi.a.23

**Published:** 2025-08-29

**Authors:** Thomas St. Pierre, Katherine S. White, Elizabeth K. Johnson, Samuel Ronfard

**Affiliations:** Institute for Language Sciences, Utrecht University, Utrecht, The Netherlands; Department of Psychology, University of Toronto Mississauga, Mississauga, ON, Canada; Department of Psychology, University of Waterloo, Waterloo, ON, Canada; Department of Psychology, University of Toronto, Toronto, ON, Canada

**Keywords:** selective learning, developmental sociolinguistics, language attitudes, exploration

## Abstract

Much of what children know about the world is learned from information provided by others, and children’s endorsement of this information depends on the social attributes of the person providing the information (e.g., their accent, attractiveness, etc.). Previous work on how the identity of a person providing information (i.e., informant) influences children’s learning has tended to focus on a highly specific, simplified learning context, where children are provided with conflicting claims from two individuals (e.g., one foreign- and one locally accented speaker) and are immediately asked to indicate whose information they endorse more. In the current study, we investigated the effect of informant identity on 5- to 7-year-old children’s (*N* = 144) learning in a more real-world context, where children encountered surprising information from only one person (a foreign- or locally accented speaker), and were subsequently given the opportunity to engage further with that information (by testing for themselves whether the information was true). In contrast to previous research using a forced choice method, almost all children initially endorsed the surprising claim; however, their subsequent testing of the claim and later endorsement *did* differ based on whether children were interacting with a foreign- or locally accented speaker. These results highlight the need to investigate the influence of social factors on selective learning in more ecologically valid contexts, which, importantly, consider the influence of an informant at multiple points throughout the learning process.

## INTRODUCTION

Imagine someone tells you that, technically speaking, bananas are berries but strawberries are not. You may make an internal judgment about how much you think their statement is actually true, but you will also overtly react to the information in some way, for example, by nodding, responding verbally (*Really?!*, *suuurrre*, *No way!*, etc.), or, in today’s world, pulling out your phone to Google the information for yourself (or secretly later). Importantly, both your evaluation of the information *and* your overt reaction to it will depend greatly on *who* the information provider is (and your relationship to them). You may, for example, more readily believe a botanist than a toddler. And, if the information source is a well-respected world leader, just nod and smile in agreement (even if you disagree) rather than pull out your phone in disbelief. Children, too, routinely encounter surprising information, and must decide—based on *who* is providing the testimony—not only how much they believe the information, but also how they will respond to and engage with that information over time. Despite the complex, multi-faceted nature of this process, previous work on how the identity of a person providing information influences children’s learning has tended to focus on a highly specific, simplified learning context, where children are provided with conflicting claims from two individuals and are immediately asked to indicate whose information they endorse more. Here, we consider how the identity of individuals providing information, as signalled by their accent (foreign or local), influences children’s learning at *multiple* points throughout the learning process, including their initial endorsement of surprising information and the extent to which they attempt to subsequently verify the truth of the information for themselves.

In the type of simplified context described above, previous research has shown that the extent to which children choose to accept what others say (e.g., that the world is round) is influenced by the characteristics of the person providing the information (e.g., Gweon, 2021; Harris et al., 2018; Tong et al., 2020). From an early age, children prefer to endorse or seek information from in-group members compared to out-group members (Dunham et al., 2008). For example, with respect to linguistic group membership (e.g., accent)—the focus of the current study—children are more likely to look to locally accented speakers (i.e., in-group members) compared to foreign-accented speakers (i.e., out-group members) when learning novel labels (Corriveau et al., 2013), the functions of novel objects (Kinzler et al., 2011), and even which foods to try (Shutts et al., 2009). Selectively attending to and learning from in-group members is thought to help children acquire information that is relevant for the groups/cultures they belong to (Begus et al., 2016; Fong et al., 2023).

However, the simplified context in which children’s learning has primarily been assessed is particular in (1) the way that information is presented to children, and (2) the way that children are able to react to this information, both of which fail to capture the breadth of situations that children find themselves in when learning from others in the real world. With respect to (1), previous work has typically shown children two individuals at a time (e.g., a foreign- and locally accented speaker), who provide conflicting pieces of information that children then need to evaluate. While this certainly happens in some contexts, in the real world, children often simply hear information from one individual rather than multiple (McDonald & Ma, 2016). In such cases, it is less clear how much the identity of an individual (e.g., their attractiveness, whether they have a foreign accent, etc.) actually influences the extent to which children believe what they say.

With respect to (2), how children respond to new information, previous work has typically asked children to simply indicate (i.e., endorse) whom (or whose information) is correct. While endorsing information provided by others can certainly indicate that children believe the information they are endorsing, this is not necessarily the case. Adults, for example, routinely signal agreement with what others say for a variety of reasons (e.g., to avoid conflict, out of deference to higher status individuals, to affiliate with others, etc.), even if they disagree. More importantly, children’s experience/engagement with information provided by others often extends beyond their immediate reaction to it (i.e., their initial decision to support it or not). While in some cases, children may fully accept what others say and quickly move on with their lives, in other cases, they may wish to seek out their own empirical evidence, ask follow-up questions, and consult additional informants as they assess the validity of a claim, all of which provide insight into how children learn from others. Only recently has work begun to consider how children’s beliefs about what others say evolve over time and influence subsequent behavior (Hermansen et al., 2021; Orticio et al., 2024), and despite the many years of work looking at how the identity of a person providing information influences children’s learning, to our knowledge, none of it has examined what happens after the initial endorsement.

In the current study, we begin to address this surprising gap in our understanding of children’s learning from others. Specifically, we investigated the influence of an individual’s accent on how children respond to a surprising (and false) piece of information, not only in their initial endorsement (or not) of the information, but also in changes in the endorsement over time as they are given opportunities to verify the truth of the claim for themselves. We focused on experimenter accent for several reasons. First, children have been shown to privilege accent over other social cues, such as race (Kinzler et al., 2009; but see Cohen et al., 2021), when evaluating and categorizing others. Furthermore, accent effects have been documented across a wide range of tasks (Kinzler, 2021) spanning both social (e.g., friendship preferences) and epistemic (e.g., selective learning) domains, and have been found in response to both culturally relevant (e.g., Fong et al., 2023; Sobel & Finiasz, 2020) and culturally irrelevant testimony about the physical world (e.g., McDonald & Ma, 2016; see also Lev-Ari & Keysar, 2010). Despite this, developmental researchers rarely report the accent of experimenters in their studies (St. Pierre et al., 2024). Given the strength and pervasiveness of accent effects, we considered experimenter accent to be a strong test case for examining the potential influence of experimenter identity on children’s endorsement and testing of claims over time.

To investigate the influence of experimenter accent on children’s learning from testimony, we used a paradigm developed by Ronfard et al. (2018). In this paradigm, children are presented with different-sized Russian dolls and provided with a surprising (and also false) claim that the smallest one is the heaviest (actually, as one would expect, the smallest is the lightest). Then, children are asked by an experimenter which one they think is the heaviest (initial endorsement), given the opportunity to test whether the claim is true after the experimenter leaves the room (testing of claim), and later asked by the experimenter again which doll is the heaviest (second endorsement), after they have acquired firsthand knowledge (if they tested) that the experimenter’s claim was incorrect (Palmquist & Kondrad, 2024). Critically, we manipulated between-subjects whether the experimenter was a foreign- or locally accented speaker of English, allowing us to assess whether children’s responses and behavior differed as a function of the language background of the informant throughout the various phases of the experiment.

With respect to children’s initial endorsement, we would expect that if children are generally more skeptical of foreign-accented speakers compared to locally accented speakers, they would be less likely to endorse surprising claims from the former compared to the latter. Alternatively, if children accept the claim regardless of speaker, or do not want to disagree with them, they may endorse the claims of foreign-accented and locally accented speakers to the same extent. However, even if children do not differ in their initial endorsements of the surprising claim, we may find that differences in children’s endorsements emerge later, after they are left alone and given the opportunity to test for themselves whether the claim is true. For example, if children are indeed more skeptical of foreign-accented speakers, they may be more likely to test the claim for themselves when interacting with foreign-accented speakers compared to locally accented speakers, and later, be less likely to endorse the claim of foreign-accented speakers. We may also find the opposite pattern of results, if, for example, children are actually more accepting of surprising/improbable information from foreign-accented speakers (see Bowman-Smith et al., 2019; Weatherhead et al., 2018). Importantly, this multi-step paradigm allows us to test whether attributes of a single person providing surprising information influence children’s behavior both in their initial endorsement of that information, and also in whether they test and subsequently update their endorsement of it. Crucially, whether children endorse and/or test surprising claims more from foreign-accented or locally accented experimenters, results would show that the identity of the person providing the information plays an important role in how children learn from others.

## METHODS

The experimenter protocol, data and syntax files, and Supplementary Materials for this study are openly available at the Open Science Framework at https://osf.io/2m4yn/. This study was approved by the ethics committee of the University of Toronto.

### Participants

We recruited 5- to 7-year-old children (*N* = 144) because this age group reliably tests surprising claims from locally accented speakers (Hermansen et al., 2021; Ronfard et al., 2018, 2021). We randomly assigned children to interact with either a locally accented (abbreviated LocA) experimenter (*n* = 71, 34 girls, *M*_*age*_ = 6.65, *SD* = 0.77), or with a foreign-accented (abbreviated ForA) experimenter (*n* = 73, 37 girls, *M*_*age*_ = 6.55, *SD* = 0.81). We tested a racially/ethnically diverse sample, reflective of the Greater Toronto Area, which is approximately 44% White, with the largest visible minority groups being of South Asian and Chinese descent (Statistics Canada, 2023). With respect to parental education, 36.11% reported that both parents had a university degree, 38.89% reported that only one parent had a university degree, and 25% reported that neither parent had a university degree.

Given the linguistic diversity in the community we drew our sample from, we also measured children’s (131/144) routine day-to-day exposure to non-local accents using a seven-point scale (see Supplementary Materials). We found no difference in accent exposure across conditions: LocA experimenter (*n* = 65), *M* = 3.28, *SD* = 1.88; ForA experimenter (*n* = 66), *M* = 3.17, *SD* = 1.65; *t* = .33, *p* = .74. Additionally, children’s accent exposure did not predict their behavior in the experiment, and is not discussed further (see Supplementary Materials for more information).

In addition to the children in our final sample, 13 other children were recruited but not included in our analyses because of equipment failure (*n* = 1), experimenter error (*n* = 5), failing to initially state that the biggest doll was the heaviest (*n* = 3), lifting the dolls before being left alone (*n* = 2), or failing to complete the experiment (*n* = 2).

### Materials

**Figure 1.**
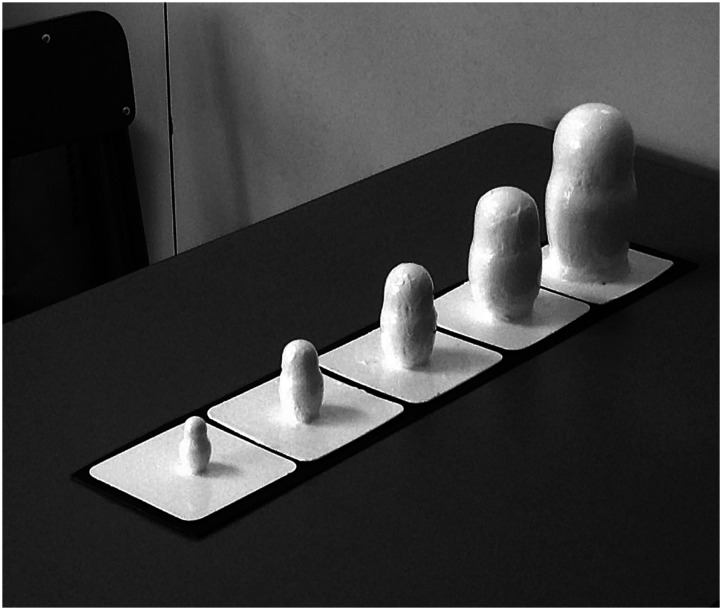
Stimuli used in the experiment.

We used five different-sized Russian nesting dolls; each was attached to a square base for stability and painted white (see Figure 1). With the square base attached, the dolls weighed (in grams): 16.32, 29.04, 46.75, 85.82, and 167.73. Crucially, the weight of the dolls aligned with what one would expect, with larger dolls being heavier than smaller ones.

### Procedure

After obtaining consent, children went into a room where the primary experimenter sat waiting at a table on which the five Russian nesting dolls stood (see Figure 1). After being greeted by the experimenter (“Hi, my name is [NAME]. What is your name?”) and given a brief introduction to the task (“Do you see these dolls here? I’m going to ask you questions about them, ok?”), children completed three phases: (1) an initial endorsement phase, where children were first presented with the surprising information from the experimenter and tested on whether they endorsed it or not, (2) an exploration phase, where children were subsequently given the opportunity to empirically test the truth of the experimenter’s claim (without her there), and (3) a subsequent endorsement phase, in which children were tested on whether they still endorsed the surprising claim of the experimenter.

At the start of the initial endorsement phase, the experimenter asked children to point to the heaviest doll, and explain their judgment. All children included in the final sample pointed to the biggest doll. Children then received counter-intuitive information from the experimenter: “Actually, that one is not the heaviest; this one [pointing to the smallest] is the heaviest. It’s heavier than all of the other ones. It’s heavier than this one, this one, this one, and this one [starting with the biggest one and moving to the others].” Note that this statement was false (the smallest doll was in fact the lightest). The experimenter again asked children to identify the heaviest doll and to explain their judgment; this served as children’s initial endorsement (or rejection) of the experimenter’s claim.^1^

At the start of the exploration phase, the experimenter told children that she was going next door to use her phone, but that she would come right back. Before leaving, she added: “Here, I’ll move the dolls closer to you,” and moved the tray to about 6 inches from the child, giving them an implicit invitation to explore the dolls while she was away.

The subsequent endorsement phase began when, after a minute, the experimenter returned and gave children an opportunity to report about their time alone in the testing room with the dolls, saying, “Okay, we’re almost done. Is there anything you want to tell me?” By doing this, children who had tested the experimenter’s claim could report that she was wrong if they wanted to. Subsequently, children were asked again by the experimenter to identify the heaviest doll by pointing (“Which doll do you think is the heaviest? Can you point to the one you think is the heaviest?”), thereby testing (again) children’s endorsement (or not) of the false claim, which may have changed if they had gathered evidence that the experimenter was wrong.

Crucially, the experimenter that children interacted with was one of four experimenters, who spoke either with a local Canadian accent (two women) or a foreign accent (one Ukrainian- or one Mandarin-accented woman). In each accent condition, one of the experimenters was white and the other was Chinese(-Canadian); preliminary analyses showed no differences between accent conditions based on experimenter race. Of interest for the current study was whether children’s endorsements of the surprising claim, and their testing of it, differed based on the accent of the experimenter.

After asking for children’s endorsement, the experimenter was replaced by a second experimenter, who also asked children which doll was the heaviest and gave them an opportunity to win a prize by putting the heaviest on a scale. We included another experimenter because we expected that if children were reluctant to openly disagree with the primary experimenter, they might be more forthcoming with a new adult who had not provided the original testimony (see Jaswal et al., 2009). However, since the accent of the second experimenter always differed from that of the primary experimenter (i.e., if the experimenter had a local accent, the second experimenter had a foreign accent, and vice versa), it was not possible to disentangle possible effects of experimenter change from those of accent. For this reason, we do not discuss this portion of the experiment further in the manuscript, but refer readers to the Supplementary Materials for a discussion.

### Coding

A research assistant blind to the hypotheses of the study coded the videos for children’s exploration of the dolls using Mangold INTERACT (Version 18.4 – 2020). A second research assistant provided reliability coding for 39 of the videos, 27% of the total sample. These research assistants coded every time a child picked up and put down each of the dolls. From these data, we extracted the following information: (1) how much time elapsed before children picked up their first doll; (2) the total number of times children picked up each doll; (3) how long (seconds) children held each doll; (4) whether children picked up the smallest doll and any other doll, as well as whether that occurred at the same time. Picking up these two dolls is the minimum amount of evidence necessary to evaluate the truth of the surprising claim. Inter-rater agreement, as measured by Cohen’s Kappa (*κ*), was excellent, *κ* = .96. Because INTERACT is time-linked, inter-rater agreement is based on the overlap between the start and end time of each coded action and is therefore applicable to all extracted measures.

## RESULTS

After being told that the smallest doll was the heaviest, the vast majority of children pointed to the smallest doll as being the heaviest, 62 out of 71 (87%) children interacting with a LocA speaker, and 63 out of 73 (86%) children interacting with a ForA speaker. Interestingly, we found no difference based on the accent of the experimenter, *χ*^2^(1) = 0.03, *p* = .86, Cramér’s *V* = .02. In the absence of competing information from multiple individuals (a LocA *and* ForA speaker), children were equally likely to accept the surprising information from ForA and LocA individuals (at least based on their initial endorsements).

However, later in the experiment, after children had been given the opportunity to explore the dolls for themselves, we *did* find differences across conditions in children’s endorsements of the surprising claim. First, when the experimenter—upon returning to the room—asked if children wanted to report anything (“Is there anything you want to tell me?”), children were slightly more likely when interacting with a LocA experimenter to explicitly say that the experimenter’s surprising claim was wrong (28%, 20 out of 71), compared to children in the ForA condition (15%, 11 out of 73), *χ*^2^(1) = 3.66, *p* = .056. A similar pattern was observed when restricting analyses to children who knew that the experimenter was wrong because they had picked up the smallest doll and one other doll: 39% (20 out of 51) vs. 24% (8 out of 35), *χ*^2^(1) = 2.53, *p* = .11.

**Figure 2.**
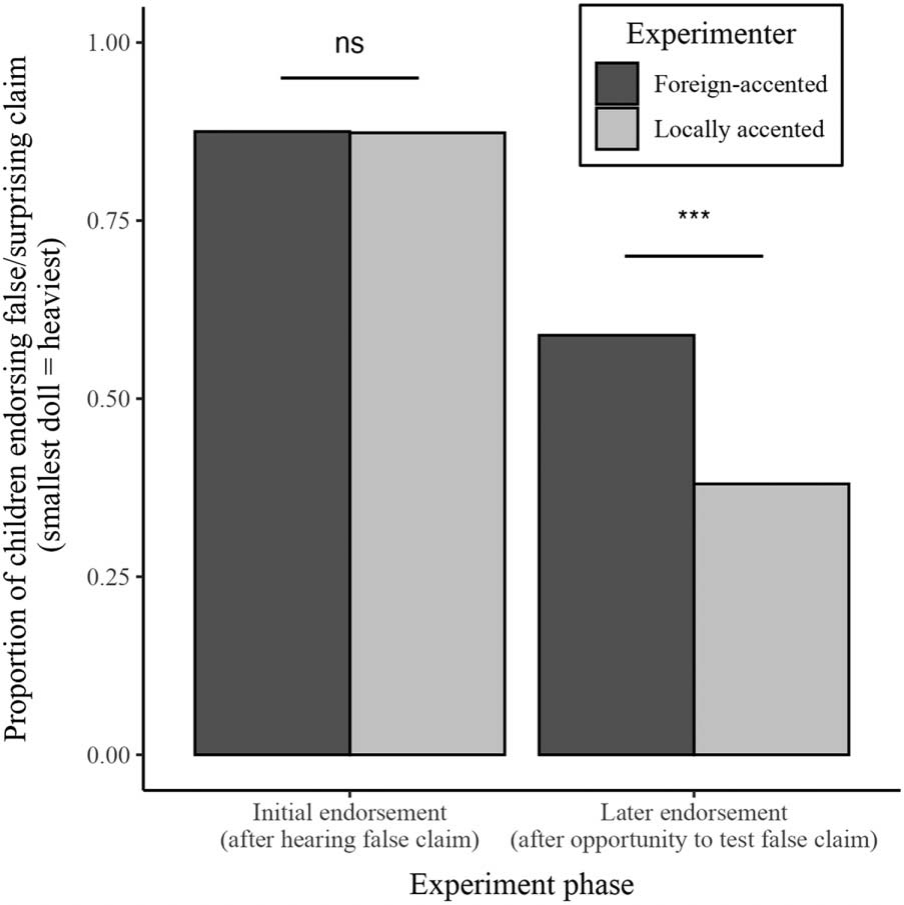
Proportion of children who endorsed the surprising and false testimony that the smallest of five Russian nesting dolls was the heaviest.

Critically, although children were overall less likely to endorse the surprising claim after the exploration phase (49%), this drop was much larger for children interacting with LocA experimenters. Specifically, while only a minority of children interacting with a LocA experimenter still endorsed the experimenter’s false claim (38%), the majority of children (59%) who interacted with a ForA experimenter still continued to *falsely* claim that the smallest doll was the heaviest (see Figure 2). A logistic regression predicting the likelihood of endorsing the surprising claim from endorsement (initial/subsequent), accent (LocA/ForA), and their interaction confirmed this pattern, revealing a marginal interaction between endorsement and accent (*z* = 1.82, *p* = .07), whereby children who interacted with a LocA experimenter were significantly less likely to endorse the surprising claim compared to children interacting with a ForA experimenter, but only after given the opportunity to explore the dolls (post-hoc tests; endorsement after exploration phase: *χ*^2^(1) = 6.64, *p* = .01; initial endorsement before exploration phase: *χ*^2^(1) = .04, *p* = .85).

**Figure 3.**
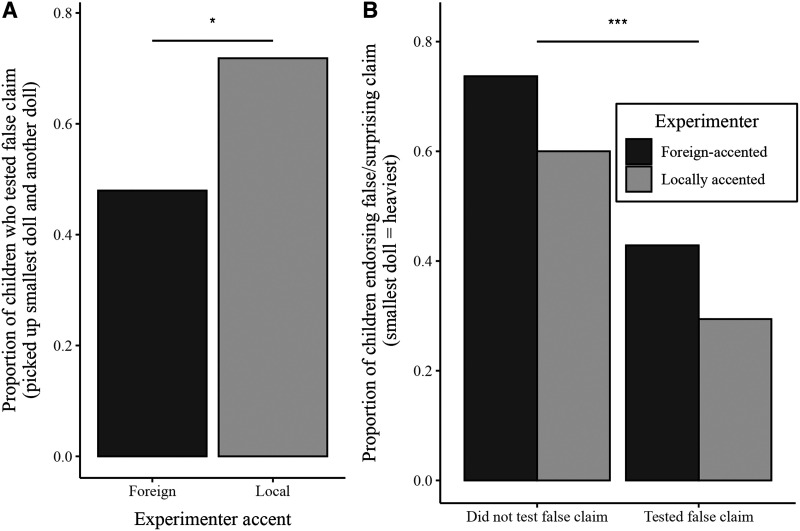
(a) Proportion of children who tested the experimenter’s claim by picking up the smallest doll and any other doll. (b) Proportion of children who endorsed the experimenter’s claim later in the experiment based on experimenter accent and whether they tested the claim themselves or not.

These large differences we observed in children’s second endorsement of the surprising information appear to hinge on whether children actually tested—during the exploration phase—whether the experimenter’s surprising claim was actually true or not (see Figure 3a). While 72% of children with a LocA experimenter (51 out of 71) tested whether the smallest doll was actually the heaviest (by picking up the smallest doll and another doll), only 48% of children with a ForA experimenter (35 out of 73) obtained such evidence^2^, a significant difference, *χ*^2^(1, *N* = 144) = 8.54, *p* = .003, Cramér’s *V* = .24. When accounting for this in children’s subsequent endorsement (or not) of the experimenter’s claim, we find that children who tested the experimenter’s claim, and knew that the smallest doll was in fact not the heaviest, were also more likely to reject the experimenter’s claim by pointing to a larger doll (see Figure 3b). A logistic regression predicting the likelihood of children endorsing the surprising claim (that the smallest doll was the heaviest) from experimenter accent (LocA or ForA), whether children tested (tested or did not test), and their interaction revealed a significant main effect of testing, with children who had explored the dolls for themselves significantly less likely to endorse the experimenter’s false claim that the smallest doll was the heaviest compared to children who did not test (*z* = 2.33, *p* = .02). While there was a tendency for children to endorse the false claim more if the experimenter had a foreign accent, this effect was not significant (*z* = 1.06, *p* = .29), nor was the interaction between experimenter accent and testing (*z* = .05, *p* = .96). This same pattern held when focusing only on children who initially endorsed the surprising claim (125/144). Thus, experimenter identity influenced children’s propensity to test the surprising claim, leading to downstream differences in how likely children were to endorse the experimenter’s claim.

## DISCUSSION

Children learn a great deal about their world through interactions with others. Does the identity of the person who provides them with new information change how readily they incorporate it into their world view? Past research suggests that when forced to choose between information provided by someone with the same accent and someone with a different accent, children are more likely to trust the same-accented individual. But in the real world, children are not always provided with two opposing world views and forced to choose. Instead, they commonly encounter information from one person alone, and choose whether to accept or reject it outright, or to seek additional evidence to test the validity of a claim. In the current study, we are the first to test not only children’s endorsements of claims by *either* a same-accented or other-accented speaker, but also how likely they are to seek additional information to test the claim and potentially reject the claim based on the evidence they collect. Using a novel design, we demonstrate that the accent of someone providing new information has repercussions for children’s learning *beyond* the first endorsement, influencing how children subsequently engage with that information over time.

In our more real-world paradigm, in which children heard a surprising claim from only one individual (rather than two conflicting claims from two individuals), we found no differences in the extent to which children initially endorsed information from a ForA speaker compared to a LocA speaker. Regardless of the experimenter’s accent, the vast majority of children endorsed the false claim that the smallest of five Russian nesting dolls was the heaviest. This suggests that the influence of accent on children’s endorsements found in previous work (e.g., Corriveau et al., 2013) may be specific to the nature of the task used, and may not generalize to more naturalistic learning contexts. Based on their endorsement behavior alone, in a situation where children hear surprising information from one individual only, they appear to accept (or reject) it, regardless of the social characteristics (e.g., accent) of the person providing it (see Vanderbilt et al., 2014).

Critically, although we found no differences in children’s initial endorsement of the surprising claim, later in the experiment, after children had the opportunity to verify whether the claim was true by picking up the dolls, we *did* find large differences in children’s endorsement. Specifically, while the majority of children who were interacting with a ForA experimenter still endorsed the false claim, only a minority of children interacting with a LocA experimenter continued to do so. This was related to the fact that significantly more children in the LocA condition actually tested whether the smallest doll was indeed the heaviest (when the experimenter left the room), and thus, had first-hand knowledge that the experimenter’s claim was untrue.

This study clearly establishes that experimenter attributes influence how children respond to a surprising claim *beyond* their initial endorsement. Now, *why* did children test the surprising claim less when interacting with a ForA experimenter compared to a LocA one? One possibility is that children’s decision to test the experimenter’s claim was related to their perception of the experimenter’s competence, and how much they considered and/or weighed the testimony of each experimenter (see e.g., Andrási et al., 2024). Even though most children initially endorsed the experimenter’s surprising claim, regardless of accent, they may have nevertheless differed in how much they actually considered the information to be true. For example, if children placed more weight on what the LocA experimenters said, they may have felt more surprised by the information (see Lane & Harris, 2015), and consequently, more compelled to test the surprising claim for themselves, compared to when the information was provided by the ForA speakers, whose statements they may have simply dismissed and not bothered to test. Alternatively, children may have tested less with ForA experimenters because they were actually *more accepting* of their surprising claim compared to LocA experimenters. Previous work, for example, has shown that children are more likely to find improbable events more possible if they occur in distant lands (Bowman-Smith et al., 2019). Given that children infer that ForA speakers are ‘not from around here’ (Kinzler & DeJesus, 2013; Weatherhead et al., 2018, 2019), live in unfamiliar looking dwellings, and wear unfamiliar looking clothes (Hirschfeld & Gelman, 1997; Wagner et al., 2014), they may have been more open to and accepting of the possibility that the dolls (which were perhaps from a distant place) might work differently than expected.

Crucially, if either of these explanations is true, it would suggest that there can be a dissociation between children’s endorsements of what others say and their true beliefs. Just as adults may routinely signal agreement with others, even if they secretly doubt them, or openly disagree with someone when they secretly agree, so too might children’s endorsement of claims be influenced by a variety of factors other than their underlying beliefs (see Jaswal & Kondrad, 2016 for a discussion). In fact, a sizeable minority of children who *had* tested the surprising claim for themselves, and therefore knew that the experimenter was wrong, *still* continued to endorse the false claim (43% of children interacting with a ForA experimenter, and 29% with a LocA experimenter). Even if children generally do agree with a claim, they will nevertheless vary in their skepticism, and the degree to which they actually think a claim is true. Thus, in order to gain a more complete and nuanced understanding of children’s underlying beliefs about testimony, and how they learn from others, it is necessary for researchers to look beyond whether children simply endorse a claim or not, and explore how their underlying beliefs might be expressed in other ways (for example, their testing of a claim).

Another possibility for why children’s testing differed by experimenter may be related to children’s perception of the experimenter’s warmth/benevolence (Fiske et al., 2007). Despite the experimenters being trained to exhibit similar expressiveness and vocal affect, children may have felt more or less comfortable with picking up the dolls, depending on the accent of the person they were interacting with. Interacting with experimenters with familiar accents in a strange laboratory setting, for example, may have put children more at ease compared to interacting with an experimenter with an unfamiliar accent; as a result, they may have felt more at liberty to pick up the dolls with LocA experimenters compared to ForA experimenters (should they have wanted to). Although not significant, compared to children interacting with a locally accented experimenter, children in the ForA condition who had tested the surprising claim were less likely to spontaneously tell the experimenter upon returning that her claim was false, suggesting perhaps that children felt less comfortable around the experimenter when she had a foreign accent. Additional evidence for this possibility comes from a recent delayed gratification study, in which children were found to wait *longer* for a second treat for out-group experimenters (differing in race and accent) compared to in-group experimenters, possibly because they felt more uncomfortable in intergroup interactions and less at liberty to touch (and eat) the immediately available treat if they wanted to (St. Pierre et al., 2023).

Finally, it is important to emphasize that the information that children received from the experimenter was actually *misinformation*, meaning that children who were still endorsing the experimenter’s claim at the end of the experiment were endorsing information that was untrue. Crucially, children were most likely to eventually reject this untrue information if they tested the veracity of the claim for themselves, demonstrating a benefit of seeking firsthand evidence when combatting misinformation (Hermansen et al., 2021; Orticio et al., 2024). However, what the current study makes clear is that the extent to which children seek out firsthand knowledge for themselves is dependent on *who* is providing the misinformation. While the relative weights of Russian nesting dolls is rather innocuous information, in real-world contexts, the proliferation of misinformation represents a significant challenge (Ecker et al., 2022). Looking at how children engage with (mis)information over time, including the factors that influence their inclination to seek out firsthand evidence for themselves, will help researchers and practitioners better understand how to prepare children to navigate our modern world.

## ACKNOWLEDGMENTS

We would like to thank Samantha Cottrell, Mariam Galytskyy, Lisa Hotson, Khrystyna Mandziy, Maryan Salad, Grace Wang, and Runyi Yao for their help with recruitment, conducting the experiment, and coding.

## FUNDING INFORMATION

This research—approved by the ethics committee of the University of Toronto—was funded by a Canadian Social Sciences and Humanities Research Council (SSHRC) grant awarded to the last author. We have no conflicts of interest to disclose.

## AUTHOR CONTRIBUTIONS

T. S. P.: Conceptualization; Investigation; Methodology; Project administration; Supervision; Writing – original draft; Writing – review & editing. K. S. W.: Conceptualization; Methodology; Writing – review & editing. E. K. J.: Conceptualization; Methodology; Project administration; Supervision; Writing – review & editing. S. R.: Conceptualization; Formal analysis; Funding acquisition; Investigation; Methodology; Project administration; Resources; Supervision; Validation; Visualization; Writing – review & editing.

## DATA AVAILABILITY STATEMENT

The data and syntax files for this study are openly available at the Open Science Framework at https://osf.io/2m4yn/.

## Notes

^1^ Children were also asked to recall which doll the experimenter had identified as the heaviest; all but 10 of the 144 children (7%) pointed to the correct doll. Our statistical conclusions hold when these 10 children, who may not have remembered the informant’s testimony, are excluded.^2^ In Supplementary Materials, we demonstrate significantly greater exploration of the dolls in the LocA than in the ForA condition across three additional measures of exploration: (1) the number of times children picked up each doll; (2) the amount of time in seconds that children held each doll; and (3) whether children picked up the smallest and the biggest doll *at the same time* to compare their weight.

## Supplementary Material


