# Supplementary material for: The Quest for Truth: Experimenter Identity Impacts Children’s Response to Surprising Information
Source: Open Mind (Camb). 2025 Aug 29;9:1363–74. doi: 10.1162/opmi.a.23 (PMC12435985; doi:10.1162/opmi.a.23)
Supplement: Supplementary file 1 [file opmi-09-1363-s001.docx]

**Supplementary Materials**

**Language questionnaire**

To measure children’s exposure to locally accented English relative to other types of accents, we asked parents to provide information about their child's exposure to other languages and English accents. Specifically, we asked them to select their child’s level of exposure using the following scale: 1 = In daily interaction, the child has almost always heard Toronto English; 2 = In daily interaction, the child has mostly heard Toronto English; 3 = In daily interaction, the child has heard a lot of Toronto English; 4 = In daily interaction, the child has heard about the same amount of Toronto-accented English as other accents of English; 5 = In daily interaction, the child has heard less Toronto-accented English than other accents of English; 6 = In daily interaction, the child has mostly heard accents of English other than Toronto English; 7 = In daily interaction, the child has since birth almost always heard accents other than Toronto English. Ninety-one percent of partents (131 out of 144) completed this survey (91%).

**Additional analyses of children’s exploration of the dolls**

*(1) The number of times children picked up each doll*

We coded the number of times children picked up each doll by condition (see Figure 1, below). We examined these data using a mixed-effect regression model with the between-subjects factor of Condition (2: locally accented vs. foreign-accented) and the within-subjects factor of Doll (5: one [i.e., smallest], two, three, four, and five [i.e., biggest]) on the square root of the number of times children picked up a doll. We took the square root of the number of times children picked each doll to meet the assumptions of regression. This analysis revealed: a main effect of Condition, χ^2^ (1) = 12.39, *p* < .001, a main effect of Doll, χ^2^ (4) = 192.04, *p* < .001, and a significant interaction between Condition and Doll, χ^2^ (4) = 13.78, *p* = .008.

As Figure 1 shows, all of the dolls were picked up more frequently by children who received testimony from a locally accented experimenter compared to children who received testimony from a foreign-accented experimenter, all *p*s < .014. In the locally accented condition, children picked up the smallest doll more frequently than all other dolls (all *p* < .001), picked up the biggest doll more frequently than the middle dolls (all *p* < .014), and picked up the middle dolls at similar rates. In contrast, in the foreign-accented condition, children picked up the smallest doll more frequently than every other doll (all *p* < .001) but picked up all other dolls at similar rates.

*Figure 1.* Number of times children in each condition picked up each doll. Error bars represent 95% confidence intervals.

*(2) The amount of time in seconds that children held each doll*

We also coded the number of seconds children held each doll by condition (see Figure 2, below). We examined these data using a mixed-effect regression model with the between-subjects factor of Condition (2: locally accented vs. foreign-accented) and the within-subjects factor of Doll (5: one [i.e., smallest], two, three, four, and five [i.e., biggest]) on the square root of the number of seconds children held a doll. We took the square root of the number of seconds children held a doll to meet the assumptions of regression. This analysis revealed: a main effect of Condition, χ^2^ (1) = 11.02, *p* < .001, a main effect of Doll, χ^2^ (4) = 317.72, *p* < .001, and a significant interaction between Condition and Doll, χ^2^ (4) = 21.86, *p* < .001.

As Figure 2 shows, all of the dolls (except the middle doll) were held for longer by children who received testimony from a native-accented speaker compared to children who received testimony from a foreign-accented speaker, all *p*s < .038. In the native-accented condition, children held the smallest doll for longer than all other dolls (all *p* < .001), held the biggest doll for longer than the middle dolls (all *p* < .001), and held the middle dolls a similar amount of time. The same pattern was observed in the foreign-accented condition: children held the smallest doll longer than all other dolls (all *p* < .025), held the biggest doll longer than the middle dolls (all *p* < .001), and held the middle dolls for a similar amount of time.

*Figure 2.* Number of seconds children in each condition held each doll. Error bars represent 95% confidence intervals.

*(3) Whether children picked up the smallest and the biggest doll at the same time to compare their weight*

As a more stringent test, we also looked across conditions at whether children differed significantly in their likelihood of conducting a more deliberate test of what they had been told, picking up the smallest doll and the biggest at the same time to compare their relative weight: 27% of children in the locally accented condition (19 out of 71) demonstrated this behavior while only 12% of children in the foreign-accented condition (9 out of 73) obtained such evidence, a significant difference, χ^2^ (1, N = 144) = 4.786, *p* = .029, Cramér’s V = .18.

**Interaction with a second experimenter**

After the primary experimenter asked children (after the exploration phase) which doll was the heaviest, they were replaced by a second experimenter, who also asked children which doll was the heaviest and gave them an opportunity to win a prize by putting the heaviest doll on a scale. The accent of the second experimenter always differed from the primary experimenter (i.e., if the experimenter had a local accent, the second experimenter had a foreign accent, and vice versa).

Our rationale for adding a second experimenter was that if children were reluctant to explicitly disagree with the primary experimenter, they might be more likely to be honest with an experimenter who did not provide the original, surprising claim (see Jaswal et al., 2009). Thus, we examined whether children who were still endorsing the experimenter’s false claim with the primary experimenter would continue to do so when interacting with another experimenter, either when directly asked or when choosing which one was the heaviest (for a prize). While children in the foreign-accented condition were more likely to stop endorsing the false claim when interacting with the second experimenter (11/37 children or 30%) compared to children in the native-accented condition (4/24 children or 17%), this difference was not significant (χ^2^ (1) = 1.34, *p* = .25). Because the second experimenter’s accent differed across conditions, it was impossible to determine the extent to which this difference (were it significant) would be due to the identity of the primary experimenter or the second experimenter.

**References**

Jaswal, V. K, Lima, O. K., & Small, J. E. (2009). Compliance, conversion, and category induction. *Journal of Experimental Child Psychology*, *102*(2), 182-195. https://doi.org/10.1016/j.jecp.2008.04.006
